# Anti-seizure medication prescription preferences: a Moroccan multicenter study

**DOI:** 10.3389/fneur.2024.1435075

**Published:** 2024-08-23

**Authors:** Yahya Naji, Wafa Hrouch, Sara Laadami, Nawal Adali

**Affiliations:** ^1^“N.I.C.E.” Research Team, “R.E.G.N.E.” Research Laboratory, Faculty of Medicine and Pharmacy, Ibn Zohr University, Agadir, Morocco; ^2^Neurology Department, Agadir University Hospital, Agadir, Morocco

**Keywords:** anti-seizure medication (ASM), epilepsy—diagnosis, prescription—preferences, multicentric study, neurologists

## Abstract

**Background:**

The management of epilepsy is mainly based on antiseizure medications (ASMs). More than 20 ASMs have been introduced in clinical practice, providing a multitude of prescription choices. To date, there are no published data on the trends in ASMs prescriptions in Morocco. Therefore, we conducted a survey among practicing neurologists in seven tertiary referral hospitals in Morocco to study the current ASMs prescription preferences and their modifying factors.

**Methods:**

Our descriptive and analytical cross-sectional study was based on a survey sent between January and April 2022 to neurologists practicing in seven tertiary referral hospitals in Morocco. Information regarding the prescription of ASMs was collected using an exploitation form and analyzed using the SPSS version 13 software.

**Results:**

Based on questionnaire responses, our results showed that Valproic acid (96.3%) and Lamotrigine (59.8%) were the two most prescribed ASMs for generalized seizure types. For focal seizure types, Carbamazepine (98.8%) and Levetiracetam (34.1%) were the most commonly prescribed drugs, whereas for combined focal and generalized seizure types, the combination of Valproic acid and Carbamazepine (38.55%) was the most prescribed. Phenobarbital was the most commonly prescribed ASM for status epilepticus (40.2%). These prescription preferences were mainly due to seizure types, cost, health insurance coverage, years of experience, and additional epileptology training (*p* < 0.05).

**Conclusion:**

Our results show a shift in the prescription of ASMs in Morocco. Similar to many other countries, valproic acid and carbamazepine are considered the first-line treatments for generalized and focal seizure types. Some factors remain as major challenges in enhancing epilepsy management in Morocco.

## Introduction

Epilepsy is a global disease with an unequal distribution. At least 50 million people worldwide suffer from epilepsy, and approximately 85% of them live in developing countries (1, 2). The incidence rate is 61.4 per 100,000 person-years; it is higher in low-income countries than in high-income countries (139.0 vs. 48.9100000 person-years) (3). The overall prevalence of epilepsy is 7.60 per 1,000 people and is higher in low-income countries (3–5). In Morocco, epilepsy represents the second motive for neurological consultation, with a global estimate revealing that nearly 700,000 Moroccans suffer from epilepsy (6). Epilepsy prevalence was estimated at 1.1% of the population according to an epidemiological study conducted in Casablanca (7).

Between 66% and 88% of patients achieve remission with ASMs (4). Since the discovery of potassium bromides as the first medical treatment for epilepsy by Locock in the 19th century, new anti-seizure medications (ASMs) have been developed (8, 9). New ASMs offer better opportunities to overcome pharmacoresistance with fewer side effects and lower potential for interaction (8, 10). Although they are more expensive than older ones, they are increasingly being used in many countries. Some ASMs are also prescribed for other indications such as psychiatric disorders, migraine, and neuropathic pain (8, 11).

However, epilepsy remains mostly untreated or inadequately treated, especially in developing countries where more than 90% of patients with epilepsy do not receive appropriate treatment (1, 12), a phenomenon known as the “epilepsy treatment gap.” This gap is greater than 75% in low-income countries, and less than 10% in high-income countries (12). Morocco is no exception in terms of the treatment gap, which is estimated to exceed 70% according to local estimates during medical campaigns in rural areas and itinerant consultations conducted in southern Morocco (7, 13). This is probably linked to several obstacles, such as the patient’s socioeconomic condition, inadequate health coverage, and the lack of neurologists in Morocco.

The factors that influence prescription preferences for ASMs by Moroccan neurologists remain unknown. This study aimed to obtain an overview of the prescription of ASMs by Moroccan neurologists and the considerations that could influence this prescribing preference.

## Subjects and methods

Our descriptive and analytical cross-sectional study was conducted anonymously on a declarative basis, targeting neurologists from seven tertiary hospitals in Morocco, who were contacted through email, social media, and the Moroccan Society of Neurology. Information regarding the prescription of ASMs was collected using an exploitation form, based on a pre-established questionnaire using Google Forms. This study was conducted between January and April, 2022.

The survey consisted of four parts with a total of 11 questions. The four parts of the survey were as follows: (1) sociodemographic information, (2) the most common type of epilepsy in practice, (3) preferences for ASM prescription, and (4) reasons for these preferences. These questions were chosen based on a literature review. We ensured confidentiality and anonymity of the participants with no ethical issues.

The collected data were analyzed using the SPSS version 13 software. The quantitative variables are presented as means, and the qualitative variables are converted to frequency (percentage). The relationship between qualitative variables was determined using the Ki 2 test, with a confidence interval (CI) of 95% and a *p*-value of <0.05, which was considered statistically significant. The exclusion criteria included incomplete forms and refusal to respond.

## Results

### Demographic characteristics

A total of 82 neurologists practicing in seven different tertiary hospitals completed the survey, with a responding rate of 54%. Sociodemographic information of the respondents can be found in Table 1.

**Table 1:** Socio-demographic characteristics of the respondents in the study.

Characteristics	Number of respondents (%)
**Age (in years)**	
≤40	34 (41)
41–50	26 (32)
≥50	22 (27)
**Gender**	
Male	36 (44)
Female	46 (56)
**Years of experience**	
0–5	20 (25)
6–10	22 (27)
>10	40 (48)
**Additional training in Epileptology**	
Yes	63 (77)
No	19 (23)

### Initiation of ASMs therapy

According to our survey, the majority of respondents prescribed ASMs for patients with generalized epilepsy seizure types, coming mainly from urban areas with low education levels (Table 2).

**Table 2:** Global socio-demographic characteristics of treated patients according to the respondents.^*^

Characteristics	Number of answers (%)
**Residence**	
Urban	61 (75)
Rural	21 (25)
**Socioeconomic level** ^**^	
Lower class	37 (45)
Middle class	45 (55)
Higher class	00 (0)
**Level of instruction**	
Illiteracy	14 (18)
Traditional Education (Holy Quran School)	06 (7)
Instructional educational institutions	62 (75)
**Knowledge about Epilepsy**	
Poor	52(64)
Good	30(36)
**Use of traditional practices**	
<50% of patients	63(77)
≥50% of patients	19(23)
**Health insurance**	
<50% of patients	58(71)
≥50% of patients	24(29)
**Seizure types**	
Focal seizures	33(40)
Generalized seizures	47(57)
Unknown	02(3)

^*^The global socio-demographic characteristics of treated patients according to the responding physicians showed that 45 of the respondents saw more patients from the middle class than from the higher class. More than 52 neurologists thought that less than 50% of their patients had health insurance. However, 57% of the neurologists thought that generalized seizures were frequently encountered. ^**^The three classes have been defined according to this Moroccan report “Bourqia R.: La stratification sociale marocaine: Note de Synthèse/2006.”

### ASMs prescription preferences

**Figure 1:**
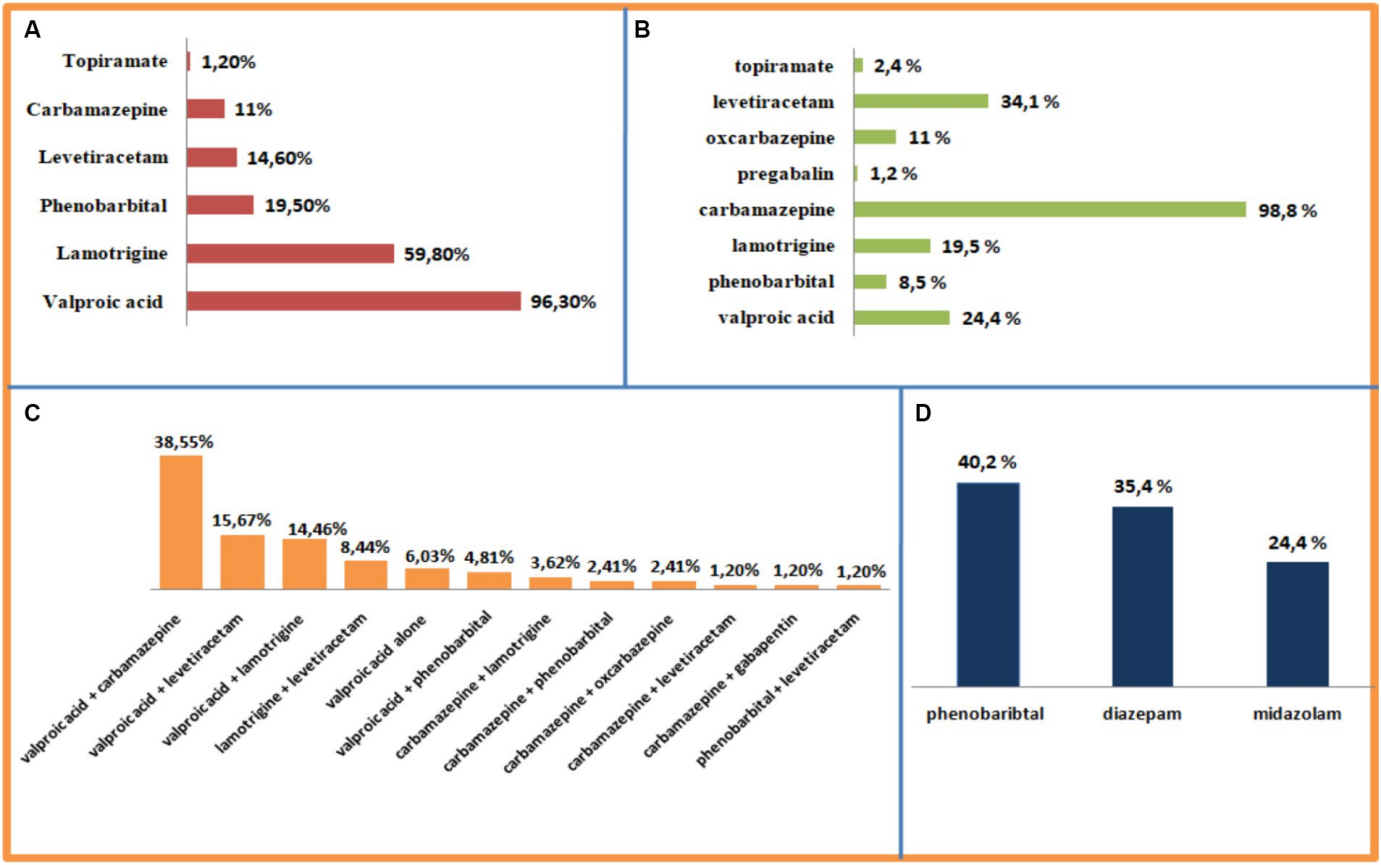
Prescription preferences for ASMs. **(A)** Generalized epilepsy. **(B)** Focal epilepsy. **(C)** Combined focal and generalized epilepsy. **(D)** Status epilepticus.

A total of 65.9% of ASM prescriptions involved monotherapy, whereas 31.1% involved polytherapy (two or more ASMs). Valproic acid (VPA) was the most prescribed ASM for generalized seizure types (96.30%), for focal seizure types, Carbamazepine (CBZ) was the most commonly prescribed ASM (98.8%). The most frequently used combination for adult patients was valproic acid/carbamazepine (38.55%), followed by Valproic acid (VPA)/Lamotrigine (LTG; 14.46%), and Levetiracetam (LEV)/Valproic acid (VPA; 15.67%). Phenobarbital (PB) was the most commonly prescribed ASM (40.2%) in patients with status epilepticus status epilepticus (SE) (Figure 1).

According to the respondents, the most critical factors in the choice of a specific ASMs for patients with epilepsy included seizure types (77% [63/82]), cost and health insurance coverage (68% [49/82]), international epilepsy treatment guidelines (57% [47/82]), unavailability of ASMs (45% [37/82]), childbearing age (44% [36/82]), potential side effects (22% [18/82]), and interactions with other drugs (20% [97/181]). The statistical significance of our study’s results, as determined by the *p*-value, underlines the main factors that affect ASMs prescription: seizure type, cost and health insurance coverage, years of experience, and additional training in epileptology. Other variables were statistically insignificant (Table 3).

**Table 3:** Variables affecting ASMs prescription and their significance level.

	Variables	Significance (*p <* 0.05)
Drug factors	Availability	0.10
	Sides effects	0.15
	Generic vs. Brand name drug	0.65
Patient factors	Seizure types	**0.02**
	Cost and health insurance coverage	**0.04**
	Childbearing age	0.52
Practitioner factors	Additional training in Epileptology	**0.04**
	Years of experience	**0.03**

Bold values indicates statistical significance, *p* value was set at <0.05.

## Discussion

In Morocco, epilepsy is the second most common cause of neurological consultation, with a global estimate revealing that nearly 700,000 Moroccans suffer from epilepsy (7). It is generally treated by neurologists, but in some cases, by neurosurgeons and psychiatrists. In 2012, there were only 120 Moroccan neurologists with a population of 31 million inhabitants, distributed unevenly, mainly in large urban centers (6, 13, 14). The median number of neurologists per 100,000 inhabitants is very low in Africa (0.03 compared to 4.5 in Europe) (15). Indisputable efforts have been devoted; today, the number of Moroccan neurologists is estimated at 240 according to the Moroccan Society of Neurology. One study has been published on types of epilepsy in Morocco, it was a local prospective study conducted over 12 months in 2011 at the university hospital in Fes, generalized epilepsies were found in 40% of consultations, of which 30% presented with generalized tonic–clonic seizures, 26% with absences, and 16% with myoclonus (16). Focal epilepsies were found in 42% of the patients, of which 60% had temporal lobe epilepsy and 10% had frontal lobe epilepsy. Unknown forms accounted for 18% of cases (16). This study partially corroborates our results, since the seizure types frequently encountered was generalized seizure types, according to 57% of the neurologists who participated in our survey, while focal seizure types came second at 40%. In Africa, only Morocco, Tunisia, Egypt, Zimbabwe, and South Africa have ASMs belonging to a new generation (6). Overall, nine ASMs are available in Morocco (Supplementary material 1), with some new ASMs, as well as galenic formulations at competitive prices.

In our study, four crucial factors determined the selection of the ASMs. Years of experience and additional training in epileptology seem to influence prescription preferences (*p <* 0.05) since they lead to more personalized treatment with new insights into the effectiveness and safety of different ASMs. Unfortunately, postgraduate training programs in neurology are only available in a few sub-Saharan African countries and are subsidized by international organizations. Thus, many neurologists have completed their training abroad (15).

Furthermore, ASMs prescription preferences depends also on seizure types (*p* < 0.05), and we noticed that ASMs prescriptions in Morocco have gradually shifted from old to new ASMs. Our results partially corroborate those of two studies conducted in Morocco in 2011, the first of which included VPA [54% were on VPA, CBZ (17%), PB (9%), and 7% on LTG (6, 16)]. Second, VPA was the most prescribed ASM (33%), followed by CBZ (31%), and PB was less prescribed (10%). Furthermore, new ASMs are being increasingly prescribed (22%) (6, 13). PB is increasingly being abandoned by Moroccan neurologists because of its significant side effects, especially in children and elder patients, while for the treatment of SE, PB remains the most prescribed in Morocco, due to the unavailability of other adapted galenic forms, such as VPA and LEV. The use of new ASMs was associated with superior efficacy in controlling SE and better functional prognosis (17, 18). A randomized controlled trial demonstrated similar efficacy of LEV and VPA in the treatment of SE in all age groups (19).

Similar prescription trends with some differences have been observed in many countries, with a considerable decrease in PB use. VPA and CBZ are the most commonly prescribed drugs in Iran (20). In China, Spain, and South Korea, LEV has increased dramatically, becoming the most commonly used ASM (21–23). In Japan, generalized epilepsy is frequently treated with VPA, and focal epilepsy with LEV (24). LTG and LEV were the most prescribed in Brazil and in England (25, 26). In Taiwan, VPA is the most prescribed ASM, Gabapentin (GBP) and Oxcarbazepine (OXC) are also frequently prescribed. The factors associated with this preference were the year of diagnosis, gender, socioeconomic status, and the presence of comorbidities (27). The most common therapeutic combinations that have been reported are VPA/LTG, LEV/OXC, and VPA/LEV in China (21) and VPA/LEV, LEV/OXC, and LEV/LTG in South Korea (23). In a study conducted in South Africa, Tanzania, Uganda, Kenya, and Ghana, only 36% of people with epilepsy received treatment, with 94.7% in PB, 40% in CBZ, and only 3.3% in VPA. These ASMs were used more for the treatment of adults (43.7%) than for children (29.0%), and were used more for focal epilepsy (45.3%) than for generalized epilepsy (31.6%), with prescriptions motivated by the presence of focal seizures, EEG abnormalities, and high seizure frequency (28).

VPA has become one of the pillars of anti-epileptic treatment, widely prescribed by many countries due to its high efficacy and its large spectrum efficacy. However, despite its tolerability and superior efficacy, its prescription must be prescribed with caution, especially in childbearing age, as exposure to VPA during the first 2 months of pregnancy is strongly associated with a wide range of malformations (29, 30). CBZ is widely prescribed for focal seizure types in Morocco, which is no longer the case in several countries because of its many serious systemic side effects, including severe allergic reactions (31), as well as the significant risk of potential drug interactions and enzyme induction (8). LEV has become the second most prescribed ASM for focal epilepsy in Morocco, which can be attributed to its exceptional pharmacokinetic profile, resulting in almost complete bioavailability, therapeutic efficacy, and low rate of side effects and drug interactions (32, 33). He does not share affinity with the targets of other ASMs, such as VPA, PB, or Diazepine, and it may also have an interesting direct neuroprotective effect (34). Nevertheless, its price has declined over the last few decades in Morocco. However, randomized controlled trials have shown that LTG is significantly more effective than LEV and CBZ for focal epilepsy (35, 36). VPA is considered a first-line treatment for generalized epilepsy; LTG and LEV would be alternatives, especially for those of childbearing age (37). Combining two ASMs with different mechanisms of action is an interesting approach for the treatment of combined focal and generalized epilepsy, allowing for better efficacy with fewer side effects. Though, only the combination of LTG and VPA has shown a synergistic interaction in clinical practice. Other possible combinations include LEV/CBZ, LEV/TPM (Topiramate), LTG/TPM, LTG/VPA, OXC/GBP, and OXC/LEV (3).

Additionally, Cost and health insurance coverage directly affected the preferences of ASMs prescription (*p* < 0.05). 62% of neurologists considered ASMs to be very expensive in Morocco compared to other countries. It should be noted that the prices of ASMs in developing countries are often higher than those of the same drugs in the United States or Europe (38), for example, the price of the same tablet of Depakine® LP 500 mg and Tegretol® 400 mg in France is on average 7,83€ and 4,59€, while in Morocco it is 105.90 MAD, 77.30 MAD, respectively (Supplementary material 2). And although efficacy and side effects (*p >* 0.05) are key factors, the choice of ASM may be more influenced by the patient’s purchasing power, especially when they do not have health insurance coverage. Previous economic evaluations have shown that the most cost-effective strategy for reducing the current burden of epilepsy in developing countries is treatment with older first-line ASMs, particularly PB (38). However, these evaluations have not guaranteed their practical application, probably due to neurologist’s reluctance to replace clinical decisions with economic influences. This hypothesis was confirmed by our study, where the majority of neurologists, prefer to prescribe ASMs such as VPA, LTG, CBZ, and LVT. In Europe, high-income countries have estimated the availability rates of ASMs to be 89% for older drugs, 88% for newer drugs, and 57% for newest drugs. In upper-middle and lower-middle income countries, availability rates were estimated at 64 and 62% for older drugs, and 58 and 40% for newer drugs, respectively, but none of the newest drugs were available in these countries (11). Among the older ASMs, it is still notable that ethosuximide is often unavailable in many countries, despite being shown to be the first-line treatment for childhood absence epilepsy (39).

## Limitations of this study

Our study has certain limitations. At first, Although the study offers an in-depth analysis of neurologists’ preferences and the factors shaping their choices, its dependence on self-reported data introduces potential biases like recall bias and social desirability bias. Additionally, the cross-sectional design restricts the ability to determine causal relationships and track changes in prescription patterns over time. Moreover, the study was limited to the main medical centers in Morocco’s, on a minor number of neurologists. Consequently, the generalizability of its findings, as they may not accurately represent the entire country.

## Conclusion

Trends in ASM prescriptions in Morocco have significantly improved, with an increase in the frequency of prescribing new ASMs and a decrease in older ones. The ASMs prescription patterns of Moroccan neurologists at seven tertiary hospitals seem to be aligned with relevant guidelines and recommendations from scientific societies. However, our results suggest that there are still many differences in ASM prescription compared to developed countries, the cost of new ASMs, their availability, and underdeveloped health insurance coverage represent an issue, which requires pharmacoeconomic evaluations to address gaps in treatment and reduce the burden of epilepsy in Morocco. The results of this survey are an illustrative reflection of the need to optimize pharmaceutical supply chains, negotiate cost-effective procurement strategies, consider local production to mitigate financial barriers, and foster collaborations between healthcare providers, government agencies, and pharmaceutical companies. Overall, a merged strategy combining medical advancements with community engagement is essential to enhance epilepsy management in Morocco.

## Data availability statement

The original contributions presented in the study are included in the article/Supplementary material, further inquiries can be directed to the corresponding author.

## Ethics statement

Ethical approval was not required for the study involving humans in accordance with the local legislation and institutional requirements. Written informed consent to participate in this study was not required from the participants or the participants' legal guardians/next of kin in accordance with the national legislation and the institutional requirements.

## Author contributions

YN: Data curation, Formal analysis, Methodology, Supervision, Validation, Visualization, Writing – original draft, Writing – review & editing. WH: Conceptualization, Investigation, Methodology, Software, Visualization, Writing – original draft, Writing – review & editing. SL: Supervision, Writing – original draft, Writing – review & editing. NA: Conceptualization, Data curation, Methodology, Supervision, Validation, Visualization, Writing – original draft, Writing – review & editing.
