# Supplementary material for: Anti-seizure medication prescription preferences: a Moroccan multicenter study
Source: Front Neurol. 2024 Aug 23;15:1435075. doi: 10.3389/fneur.2024.1435075 (PMC11378524; doi:10.3389/fneur.2024.1435075)
Supplement: Supplementary file 1 [file Table_1.docx]

**Anti-seizure medication prescription preferences:**

**A Moroccan multicenter study**

***Supplementary material***

**-1:** ASMs in Morocco until 2022:

| Available ASMs | Unavailable ASMs |
| --- | --- |
| \| **DCI** \| **Brand name drug** \| **Generic** \| \| --- \| --- \| --- \| \| Phenobarbital \| Gardénal® \|  \| \| Valproic acid \| Dépakine® \| Micropakine LP ®, Dépakine chrono®, Valprocooper LP® \| \| Carbamazépine \| Tégrétol® \| Crizépine®,Alepsia® carbamazépine Normon®, zeptol® \| \| Gabapentine \| Neurontin® \| Gabamox®, Nupentin® \| \| Lamotrigine \| Lamictal® \| Synnax® \| \| Topiramate \| Topiramate GT® \|  \| \| Oxcarbazepine \| Trileptal® \|  \| \| Levetiracetam \| Keppra® \|  \| \| Pregabalin \| Lyrica® \| Alyse®, Gabline®, Edgar®, Epilab®,Epyca ®,Gaphine ® \| | Ethosuximide  Phenytoin  Vigabatrin  Tiagabine  Acétate d'eslicarbazépine  Rufinamide  Zonisamide  Lacosamide  Stiripentol  Rétigabine  Brivaracetam |

**-2:** ASMs costs in Morocco*****:

| ASMs | Pharmaceutical dosage form | Price |
| --- | --- | --- |
| Phenobarbital (PB) | GARDENAL® 40 MG / 2 ML  GARDENAL® 50 MG Cp | 77.30 MAD  11.30 MAD |
| Valproic acid (VPA) | DEPAKINE CHRONO® 500 MG Cp  MICROPAKINE® LP 500 MG sachet | 105.90 MAD  164.80 MAD |
| Carbamazepine (CBZ) | TEGRETOL® CR 400 MG CP  CARBAMAZEPINE NORMON® 400 MG Cp  ZEPTOL® LP 400 MG Cp | 77.30 MAD  35.60 MAD  60.70 MAD |
| Gabapentin (GBP) | NEURONTIN® 300 MG, Gélule  GABAMOX® 400 MG, Gélule  NUPENTIN® 400 MG, Gélule | 287.00 MAD  155.00 MAD  114.00 MAD |
| Lamotrigine (LTG) | LAMICTAL® 25 MG Cp  SYNNAX® 25 MG Cp  SYNNAX® 100 MG Cp | 73.00 MAD  63.50 MAD  183.20 MAD |
| Topiramate (TPM) | TOPIRAMATE GT® 100 MG Cp | 187.10 MAD |
| Levetiracetam (LEV) | KEPPRA® 500 MG Cp | 524.00 MAD |
| Oxcarbazepine (OXC) | TRILEPTAL® 600 MG Cp | 335.00 MAD |
| Pregabalin (PGB) | ALYSE® 150 MG Gélule  EPIGAB® 300 MG Gelule  EPYCA® 150 MG Gélule  GABLINE® 150 MG Gélules  GAPHINE® 150 MG Gélule | 318.00 MAD  481.00 MAD  99.50 MAD  317.00 MAD  262.00 MAD |

*****According to the website [www.medicament.ma](http://www.medicament.ma)

**-3:** Google Forms Questionnaire link: <https://docs.google.com/forms/d/1Z1yFig0DlFz_htiQ_cZnEM-OIcjnBts70apQQCoF9G0/edit?ts=65a3ed38>
